# Patterns of acute chest pain at two tertiary centres in Accra, Ghana

**DOI:** 10.4314/gmj.v58i3.8

**Published:** 2024-09

**Authors:** Alfred Doku, Tom A Ndanu, Frank Edwin, Kow Entsua-Mensah, John Tetteh, Aba Ghansah, Bernard Yeboah-Asiamah, Desrie Gyan, Innocent Adzamli, Mohammed A Sheriff, Mark Tettey

**Affiliations:** 1 National Cardiothoracic Centre, Korle-Bu Teaching Hospital, Accra, Ghana; 2 Department of Medicine, Korle-Bu Teaching Hospital, Accra, Ghana; 3 University of Ghana Medical School, Accra, Ghana; 4 Community and Preventive Dentistry, University of Ghana Dental School, Accra, Ghana; 5 School of Medicine, University of Health and Allied Sciences, Ho, Ghana; 6 Department of Community Health, University of Ghana Medical School, Accra, Ghana; 7 Weija Gbawe Municipal Hospital, Accra, Ghana; 8 Department of Environmental Health and Sanitation Education, University of Education, Winneba, Ghana

**Keywords:** life-threatening, acute-onset, acute coronary syndrome, acute chest syndrome, emergency

## Abstract

**Objective:**

To assess the clinical presentation and evaluation of acute life-threatening chest pain in Accra, Ghana.

**Design:**

This was a cross-sectional study at the emergency departments of two leading tertiary hospitals in Accra.

**Settings:**

The study was conducted at the Korle-Bu Teaching Hospital and the 37 Military Hospital in Accra

**Participants:**

The study participants comprised adult patients aged 18 years and above who presented with acute chest pain at the emergency departments between April and June 2018.

**Main Outcome:**

Acute coronary syndrome is the leading life-threatening cause of chest pain with poor pre- and in-hospital care.

**Results:**

232 patients with chest pain were enrolled as respondents aged 18 to 94 years. The prevalence of life-threatening conditions causing chest pain was 31.9% of those who presented with acute chest pain. These included acute coronary syndrome (82.4%), pulmonary embolism (14.9%), and acute chest syndrome (2.7%). A few (6.6%) with life-threatening conditions such as acute coronary syndrome were transported by ambulances, and 44.3% reported to the facility within 2 to 9 days after the onset of chest pain. None of the patients with pulmonary embolism and acute chest syndrome had computer tomography pulmonary angiogram (CTPA) and echocardiogram done, respectively.

**Conclusion:**

Our study found that life-threatening conditions amongst patients presenting with acute chest pains are common; however, there is a need to improve pre-hospital care and in-hospital assessment of these cases.

**Funding:**

The study was partly funded by the Medtronic Foundation.

## Introduction

It is estimated that 25% of the general population experience chest pain in some form during their lifetime.^1^ Although the majority of patients with chest pain follow a benign course, in a smaller fraction, life-threatening conditions causing chest pain result in a fatal outcome.^2^ In low- and middle-income countries, over 80% of deaths are related to cardiovascular diseases (CVD), with chest pain being a common presenting symptom.^3^ Most of the risk factors for CVD, such as obesity, diabetes, hypertension and dyslipidaemia, are also associated with cardiac causes of chest pain.^4^,^5^

Physicians generally consider life-threatening conditions as the first differential diagnosis when patients present with acute chest pain to the emergency unit.^5^ Typical conditions entertained in such settings include acute coronary syndrome (ACS), acute aortic dissection, pulmonary embolism (PE), tension pneumothorax, oesophageal rupture, and cardiac tamponade.^6^

Acute chest syndrome (AChS) and hypertensive heart disease (HHD) are also important causes of life-threatening chest pain in the African setting.^7^ An AChS is one of the leading reasons for hospital visits among sickle cell disease patients; it also accounts for many mortalities among the sickle cell disease population.^8^ For patients with HHD, chest pain may be due to high afterload, increased left ventricular pressure and wall tension, as well as hypertrophy, which causes coronary supply-demand mismatch.^9^

A good history and physical examination constitute the foundation of the clinical diagnosis in chest pain.^10^ Many symptoms and signs, as well as diagnostic tests, have been defined for life-threatening conditions associated with acute-onset chest pain to predict the most likely diagnosis in any particular patient promptly.^11^ Nonetheless, no proper emergency response systems or pre-hospital care are available to manage these patients in resource-poor settings.^12^ Hence, late presentation often delays care for these patients. Time is very important in the management of the causes of acute onset chest pain.

To the best of our knowledge, the frequency of the causes of life-threatening chest pain has not been determined in the Ghanaian setting. The diagnostic probability that could be inferred from such data is of paramount importance in the clinical evaluation of such patients and useful in limiting needless and expensive diagnostic testing among the population with chest pain of non-life-threatening aetiology, especially in low-resource settings such as Ghana. In this study, we sought to assess life-threatening causes of acute onset chest pain in Accra, as well as the clinical presentation and evaluation by the clinicians.

## Methods

### Study Settings and Design

This was a cross-sectional study of patients presenting with chest pain at the emergency departments of two leading tertiary hospitals, the Korle-Bu Teaching Hospital and the 37 Military Hospitals in Accra, Ghana. The Korle-Bu Teaching Hospital is a 2,000-bed facility with an outpatient attendance of 1,500 per day (https://kbth.gov.gh/). The 37 Military Hospital is a 400-bed facility with an average daily outpatient attendance of 550 (https://www.ghana.gov.gh/mdas/ee0f1b1bcc/). The two hospitals offer mostly secondary and tertiary level services and serve the Greater Accra Region, other parts of Ghana, and the West African sub-region. The Korle-Bu Teaching Hospital provides 24-hour accident and emergency services that attend to all trauma, medical and surgical emergencies (referred and non-referred cases) and is the main entry point for in-patient admissions to other hospital departments. The 37 Military Hospital is the National Emergency and Disaster Hospital and has a medical emergency unit serving the enlisted personnel and the general population.

### Data collection

The study recruited consenting adult patients presenting with acute onset chest pain; chest pain within 48 hours as the primary point of call or longer as a secondary point of call characterised as central, precordial or retrosternal chest pain, which may present as chest tightness, heaviness, squeezing, gripping or chest soreness and considered to require immediate medical attention and/or intervention, as the main complaint at the emergency departments of the two hospitals from April to June 2018. Consecutive adult patients aged 18 years and above presenting with acute chest pain were enrolled in the study. After patient stabilisation, a structured questionnaire was administered by a research assistant to eligible participants.

The data extraction tool captured additional data regarding clinical evaluation information used in making the diagnosis, such as relevant history (relevant symptoms, medical history, CVD risk factors, current medication, etc.), physical examination findings (haemodynamic parameters, temperature, respiratory rate, chest examination findings) and diagnostic investigations (e.g. ECG, chest x-ray, echocardiogram, cardiac biomarkers such Troponin/CK-MB, D-Dimer, full blood count, endoscopy, CT scan, coronary angiogram, etc) and the final diagnosis determined by the attending physicians based on history, physical examination and investigations. The conditions or diseases causing the chest pain were classified as cardiac or non-cardiac causes. Cardiac causes of chest pain included stable coronary artery disease/chronic coronary syndrome, acute coronary syndrome, pulmonary embolism, pericarditis, aortic dissection, etc. Non-cardiac causes of chest pain included acute chest syndrome, musculoskeletal chest pain, peptic ulcer, gastro-oesophageal reflux disease, etc. In addition, the conditions causing chest pain were classified as life-threatening or non-life-threatening after the final diagnosis was determined.

- The definition of life-threatening acute chest pain is Chest pain caused by any disease or condition which, if the disease process is not interrupted, can lead to death (from cardiac arrest, respiratory arrest, sudden cardiac death, etc), or severe acute disability (cardiogenic shock, heart failure, obstructive shock, respiratory failure, etc). Examples of diseases causing life-threatening chest pain include acute coronary syndrome, pulmonary embolism, acute chest syndrome, aortic dissection, tension pneumothorax, etc.
- The definition of life-threatening conditions/diseases was
  - Acute coronary syndrome: Acute myocardial infarction or unstable angina
  - Pulmonary embolism: The obstruction of the pulmonary artery or one of its branches by a thrombus, air or other substances.
  - Acute chest syndrome: Vaso-occlusive crisis of the pulmonary vasculature seen in sickle cell anaemia.

**Diagnostic criteria:**

- Acute coronary syndrome
  New or worsening chest pain, ECG changes (ST-changes, dynamic T-wave changes, etc), evidence of cardiac biomarker release (in the case of myocardial infarction) or confirmed by coronary angiogram
- Pulmonary embolism
  Chest pain, dyspnoea, tachycardia, high predictive score, high D-Dimer, supportive evidence from ECG/echocardiogram or confirmed by CT pulmonary angiogram
- Acute chest syndrome:
  Sickle cell anaemia with chest pain, cough, fever (Temperature > 38.5°C), tachypnea, hypoxemia; dyspnoea, wheezing, crackles and a new pulmonary infiltrate on chest x-ray that involves at least one lung segment and is not due to atelectasis.

The study excluded patients with traumatic chest pain or known metastatic disease.

### Statistical Analysis

Data from the questionnaire and clinical information were entered into Microsoft Excel 2013 and imported to STATA version 14 for analysis. Descriptive statistics, including frequencies and proportions for categorical variables, were performed. Sociodemographic characteristics, characteristics of chest pain, diagnoses and relevant diagnostic tests were presented as frequencies and proportions in tables. Univariate data analysis involving a chi-square test was performed to assess the significance of differences in cardiac causes of chest pain between sociodemographic characteristics. A p-value of less than 0.05 was considered statistically significant.

### Ethical considerations

The Protocol and Ethics Review Committee of the College of Health Sciences, University of Ghana (CHS-Et/M.11-P4.4/2016-2017), the Institutional Review Boards of Korle-Bu Teaching Hospital (KBTH-IRB 00089/2017) and 37 Military Hospital (GHQ/5197/G/DI/2308) approved the study. Informed consent was obtained after the aim of the study was thoroughly explained to the participants.

## Results

### Patients presenting with acute chest pain at the emergency departments

Overall, 232 out of the 4,607 emergency admissions at the two tertiary hospitals presented with acute chest pain. This represents 5% of all emergency admissions during the study period. The age of the patients ranged between 18 and 94 years. More than half of the patients were females (51.7%) and resided in urban areas (57.3%). About 46.6% of them were self-employed. No significant differences were found between the causes of chest pain and the respondents' sociodemographic characteristics (p > 0.05) (Table 1)

**Table 1:** Patients presenting with acute chest pain at the emergency departments

Variable		Causes of chest pain			
	Total (n=232)	Non-Cardiac (n=132)	Cardiac (n=100)	χ^2^	p-value
Sex				0.36	0.550
Male	112 (48.3)	66(50.0)	46(46.0)		
Female	120 (51.7)	66(50.0)	54(54.0)		
Age group				2.22	0.900
<20	12(5.2)	6(4.5)	6(6.0)		
20-29	44(19.0)	25(18.9)	19(19.0)		
30-39	38(16.4)	20(15.2)	18(18.0)		
40-49	46(19.8)	26(19.7)	20(20.0)		
50-59	40(17.2)	23(17.4)	17(17.0)		
60-69	31(13.4)	21(15.9)	10(10.0)		
70+	21(9.1)	11(8.3)	10(10.0)		
Place of residence				2.22	0.329
Rural	12(5.2)	9(6.8)	3(3.0)		
Semi-urban	87(37.5)	51(38.6)	36(36.0)		
Urban	133(57.3)	72(54.5)	61(61.0)		
Occupation				9.48	0.050
Unemployed	21(9.1)	9(6.8)	12(12.0)		
Self-employed	108(46.6)	58(43.9)	50(50.0)		
Civil servant	60(25.9)	35(26.5)	25(25.0)		
Retired	20(8.6)	17(12.9)	3(3.0)		
Student	23(9.9)	13(9.8)	10(10.0)		

Data are presented as frequencies with percentages in parentheses; significant at p < 0.05; Ref=reference category

### Distribution of life-threatening conditions by sociodemographic characteristics of patients with chest pain

The study found (n=74, 31.9%) of patients with life-threatening conditions out of the total (n=232) presented with acute chest pain. These are ACS (82.4%), PE (14.9%) and AChS (2.7%). Patients presenting with ACS were predominantly males (70.5%), aged between 40-59 years (55.8%), had tertiary education (34.4%), self-employed (49.2%) and earned monthly income of more than 1000 Ghana Cedis (36.0%). A few (6.6%) with ACS were transported by ambulances, and 44.3% reported to the facility within 2 to 9 days after the onset of chest pain. Similarly, patients who presented with PE were mostly males (63.6%), aged between 40-49 years (27.3%), had tertiary education (63.6%), were employed as civil servants (45.5%) and earned a monthly income between 500-1000 Ghana Cedis (27.3%). All patients with PE were transported to the facilities by other means of transport rather than an ambulance. Most of the patients (63.6%) reported to the facility within 2 to 9 days after the onset of chest pain. All cases of AChS in the study were males, reported within 2 to 9 days after the onset of chest pain, and 50.0% of each was transported to the facilities by ambulance or other means of transport (Table 2).

**Table 2:** Distribution of life-threatening conditions by sociodemographic characteristics of patients with chest pain

Variable	Life-threatening conditions, n (%)		
	ACS (n=61)	PE (n=11)	AChS (n=2)
**Sex**			
Male	43(70.5)	7(63.6)	2(100)
Female	18(29.5)	4(36.4)	0(00)
**Age group**			
<20	1(1.6)	0(0.0)	0(0.0)
20-29	4(6.6)	2(18.2)	1(50.0)
30-39	6(9.8)	2(18.2)	0(0.0)
40-49	17(27.9)	3(27.3)	1(50.0)
50-59	17(27.9)	2(18.2))	0(0.0)
60-69	10(16.4)	1(9.1)	0(0.0)
70+	6(9.8)	1(9.1)	0(0.0)
**Educational level**			
None	6(9.8)	1(9.1)	0(0.0)
Primary	16(26.2)	2(18.2)	1(50.0)
Secondary	18(29.5)	1(9.1)	1(50.0)
Tertiary	21(34.4)	7(63.6)	0(0.0)
**Occupation**			
Unemployed	9(14.8)	0(0.0)	0(0.0)
Self-employed	30(49.2)	4(36.4)	2(100)
Civil servant	17(27.9)	5(45.5)	0(0.0)
Retired	3(4.9)	2(18.2)	0(0.0)
Student	2(3.3)	0(0.0)	0(0.0)
**Monthly income (Ghana Cedis)**			
None	15(24.6)	2(18.2)	0(0.0)
<500	15(24.6)	2(18.2)	0(0.0)
501-1000	9(14.8)	3(27.3)	1(50.0)
1001-2000	11(18.0)	1(9.1)	1(50.0)
2000-5000	11(18.0)	3(27.3)	0(0.0)
**Means of transport**			
Ambulance	4(6.6)	0(0.0)	1(50.0)
Any other	57(93.4)	11(100)	1(50.0)
**The time interval between onset and presentation**			
Within 24 hrs	18(29.5)	3(27.3)	0(0.0)
2-9 days	27(44.3)	7(63.6)	2(100)
≥10 days	16(26.3)	1(9.1)	0(0.0)
Data are presented as frequencies with percentages in parentheses; ACS: Acute Coronary Syndrome; PE: Pulmonary Embolism; AChS: Acute Chest Syndrome			

### Life-threatening conditions and the investigations requested among patients with life-threatening causes of chest pain

Table 3 shows the investigations requested among patients with life-threatening causes of acute chest pain. Almost all the patients with acute coronary syndrome had an ECG (98.4%), whereas 37.7% and 8.2% had an echocardiogram and coronary angiogram done, respectively. All patients with pulmonary embolism had ECG done; more than half of them took CXR (90.9%) and echocardiogram (63.6%), but none had a CT PA scan. All patients with acute chest syndrome had ECG and CXR done, but none had an echocardiogram done. Regarding ACS, 31.1%, 60.7% and 88.5% did not receive requests for troponin, echocardiogram and coronary angiogram.

**Table 3:** Life-threatening conditions and the investigations requested among patients with life-threatening causes of chest pain

Diagnosis	Test	Status, n (%)		
		Done	Could not afford	Not requested
**Acute Coronary Syndrome**				
	FBC and ESR	52(85.3)	0(0.0)	9(14.7)
	ECG	60(98.4)	0(0.0)	1(1.6)
	CXR (AP/LATERAL)	38(62.3)	1(1.6)	22(36.1)
	Troponin (I/T)	40(65.6)	2(3.3)	19(31.1)
	Echocardiogram	23(37.7)	1(1.6)	37(60.7)
	Coronary Angiogram	5(8.2)	2(3.3)	54(88.5)
**Pulmonary Embolism**				
	FBC and ESR	11(100)	0(0.0)	0(0.0)
	ECG	11(100)	0(0.0)	0(0.0)
	CXR (AP/LATERAL)	10(90.9)	0(0.0)	1(9.1)
	D-Dimer	5(45.5)	0(0.0)	6(54.5)
	Troponin (I/T)	9(81.8)	0(0.0)	2(18.2)
	Echocardiogram	7(63.6)	0(0.0)	4(36.4)
	CT PA Scan	0(0)	0(0.0)	11(100)
**Acute Chest Syndrome**				
	FBC and ESR	2(100)	0(0.0)	0(0.0)
	ECG	2(100)	0(0.0)	0(0.0)
	CXR (AP/LATERAL)	2(100)	0(0.0)	0(0.0)
	Troponin (I/T)	0(0.0)	0(0.0)	2(100)
	D-Dimer	0(0.0)	0(0.0)	2(100)
	Echocardiogram	0(0.0)	0(0.0)	2(100)
	FBC and ESR	2(100)	0(0.0)	0(0.0)

FBC: Full Blood Count; ESR: Erythrocytes Sedimentation Rate; ECG: Electrocardiogram; CXR= Chest X-Ray; AP: Anteroposterior; CTPA: Computer Tomography Pulmonary Angiogram.

## Discussion

The study aimed to evaluate the clinical presentation and assessment of life-threatening chest pain in Accra. The study found the prevalence of life-threatening causes of chest pain to be 31.9%. In comparison, a study in the United States of America had a prevalence of 5.9% of life-threatening conditions as the cause of chest pain.^13^ ACS was the commonest life-threatening cause of chest pain identified in this study, consistent with the finding of the National Study of Emergency Departments in the United States, where ACS accounted for 5.2% of life-threatening condition diagnoses made.^13^ In this study, the patients presenting with life-threatening causes of chest pain were predominantly males and were aged 40 years and above.

The high proportion of life-threatening cases of chest pain in this study could be because both institutions are the main referral centres in a large metropolitan urban community with functioning emergency rooms at the time of our study. Compared to similar findings on the African continent, a study in Pretoria, South Africa, found ACS as the leading cause of chest pain, accounting for 53.2% of life-threatening chest pain.^14^. Even though atherosclerotic CVD risks (e.g. hypertension, diabetes, obesity) are increasing in Africa, in general, ACS is not the leading cause of life-threatening chest pain in Sub-Saharan as shown in the study in Tanzania where heart failure (including hypertensive heart diseases) and pulmonary tuberculosis were the main causes of acute chest pain.^15^ A larger study in Ghana and Africa is needed to find the leading cause of life-threatening chest for appropriate interventions.

The study found that ECG was done in almost all the patients with life-threatening chest pain. However, CXR was not requested in approximately 36.1% of the patients with ACS. Regarding investigations and diagnosis of ACS, it has been recommended that in addition to a quick but thorough assessment of the patient's history and findings on physical examination, electrocardiography, chest radiology, and cardiac biomarker tests are required for accurate diagnosis and help in early risk stratification, which is essential for guiding treatment.^16^ For the ECG, this was consistent with our findings.

This study found that almost one-third (31.1%) of the patients did not request cardiac troponins. The study further revealed that the patients with ACS did not request an echocardiogram (60.7% of participants) or a coronary angiogram (88.5%). Cardiac biomarkers are recommended for all patients with chest pain or other symptoms suggestive of ACS because the cardiac-specific troponins T and I are highly accurate, sensitive, and specific determinants of myocardial injury.^16^ This is at variance with our findings. Regarding imaging, these were not routinely done (echocardiogram and coronary angiogram) because these are expensive diagnostic procedures in our setting. As found in this study, most of the patients earn an average monthly income equivalent to $200 or less and may not be able to make an out-of-pocket payment required for these tests to be done since such tests are not covered under the National Health Insurance Scheme. In addition, the late presentations in our study population and the type of ACS could have influenced physician decisions regarding whether to request a coronary angiogram. Furthermore, there may be low physician awareness of the availability of these diagnostic tools in the country, especially cardiac catheterisation, which was available in only two health facilities in the study setting, Accra, at the time of the study.^17^ Currently, there are six functional cath labs in Accra.

Regarding the investigation and diagnosis of PE, the study found that ECG was performed in all the patients, and echocardiography was performed in 63.6% of the patients. Transoesophageal echocardiography has been documented to allow direct visualisation of a thrombus in the pulmonary artery.^18^ However, according to the European Society of Cardiology Guidelines (2019), the diagnosis of PE may be accepted in a highly unstable patient setting based on transthoracic echocardiographic findings.^19^ Computed tomographic pulmonary angiogram (CTPA) is currently the gold standard for diagnosing PE.^20^ None of the patients with suspected PE had a CTPA in this study. This was probably due to the unavailability of this service at the two health facilities included in the study or the patients' instability at the time of presentation. It could also be related to cost, clinician unawareness, non-functional equipment, etc.

The study established that almost all the patients with AChS had ECG and CXR as part of the investigations. This is in line with the established practice where patients suspected of AChS with chest pain are required to have an ECG to exclude ST-segment elevation myocardial infarction (STEMI) and other high-risk changes.^21^ A CXR must identify radiological signs of acute heart failure (AHF) and possible alternative aetiology for the presenting dyspnoea.^22^ However, an echocardiogram, which is required in all patients with acute left ventricular failure (ALVF), and cardiac troponins, which are useful for detecting ACS as the precipitator of AHF and a surrogate for poorer outcomes in AHF19, were not done for all with AChS. This may be due to the financial burden associated with these investigations.

The study found that only 6.6% of those presenting with life-threatening conditions such as acute coronary syndrome were transported by ambulances, which is lower than the 37% reported in Pretoria, South Africa.^14^ In Ghana, there are reports of low public patronage of ambulance services.^12^ Mould-Millman and colleagues have established that in Accra, most people prefer a taxi to an ambulance in medical emergencies. They perceive commercial taxis as easily accessible compared to ambulances.^12^ Even though the National Ambulance Service has improved pre-hospital trauma, especially road traffic accidents, response/care^23^ Referral transport and pre-hospital response to non-trauma cases, especially to homes and offices, are largely non-existent. This could explain the observation made in this study. Other reasons for low ambulance usage in our survey could also be perceived cost implications, availability of ambulances in the patients' vicinity, knowledge of how to access the service, etc.

The study also found that about 70% of the patients with ACS reported to the health facilities after 24 hours of chest pain onset. This could be due to a lack of public awareness of the importance of chest pain. Another reason is that the pharmacy is the first point of call for most patients in Ghana.

Another reason is that the pharmacy is less costly and within walking distance compared to reporting to the hospital.^24^ Patients will usually report to the hospital only when the symptoms persist or worsen after taking the over-the-counter drugs bought at the community pharmacies.^24^

Low-income levels and the low availability of some relevant diagnostic tools may pose a challenge in having full diagnostic evaluation and therapy for life-threatening conditions causing chest pain. Ghana should have national policies to guide the management of emergencies, especially cardiac emergencies, where prompt interventions are crucial. These policies may cover pre-hospital care because of the poor uptake of ambulance transport in our study and funding, infrastructure, and human resource development in managing emergencies.

The National Cardiovascular Diseases Management Guidelines 2019 and their training manuals/guides should be widely used for training in pre-service (medical schools and nursing colleges) and in-service (i.e., continuous professional development) to improve cardiovascular emergency management. Further research is needed to understand better the incidence and distribution of acute chest pain in Ghana because some of the findings, such as the ages of the participants with ACS, are inconsistent with current literature.

### Study limitations

The study was conducted in two tertiary-level facilities. Hence, the findings cannot be generalised to the Greater Accra Region, country, and all levels of care, but they could be considered indicative of similar settings. The study's small sample size limits the generalisation to the larger public. The study period was only three months, and the study did not cover the type of treatment received and outcomes.

## Conclusion

The study has demonstrated that life-threatening causes of chest pain are not uncommon in the emergency departments of the two tertiary facilities in Ghana. Common life-threatening conditions identified included Acute Coronary Syndrome, Pulmonary Embolism and Acute Chest Syndrome. ECG and CXR were the common investigations done among patients. Most patients were delayed in reporting to the health facilities after the onset of chest pain, mostly after 24 hours. They were transported to the hospital utilising transport other than an ambulance. There is a need to create public awareness of chest pain, build the capacity for pharmacists to refer chest pain cases and make ECG available at lower-level facilities.

## Figures and Tables

**Figure 1:**
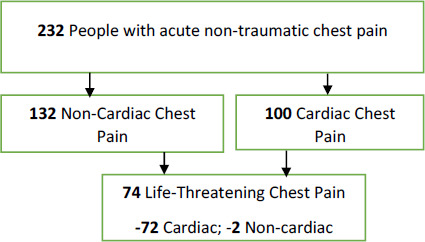
Flowchart showing acute non-traumatic chest pain
